# 2-Ethyl-6-(2-pyrid­yl)-5,6,6a,11b-tetra­hydro-7*H*-indeno[2,1-*c*]quinoline

**DOI:** 10.1107/S1600536810005805

**Published:** 2010-02-20

**Authors:** Arnold R. Romero Bohórquez, Vladimir V. Kouznetsov, Teresa González, Alexander Briceño

**Affiliations:** a Laboratorio de Química Orgánica y Biomolecular, Escuela de Química, Universidad Industrial de Santander, Apartado 678, Bucaramanga, Colombia; b Centro de Química, Instituto Venezolano de Investigaciones Científicas (IVIC), Apartado 21827, Caracas 1020-A, Venezuela

## Abstract

The title compound, C_23_H_22_N_2_, was obtained using the three-component imino Diels–Alder reaction *via* a one-pot condensation between anilines, α-pyridine­carboxy­aldehyde and indene using BF_3_·OEt_2_ as the catalyst. The mol­ecular structure reveals the *cis*-form as the unique diastereoisomer. The crystal structure comprises one-dimensional zigzag ribbons connected *via* N—H⋯N hydrogen bonds. C—H⋯π inter­actions also occur.

## Related literature

For background to polycyclic quinoline derivatives, see: Denny & Baguley (2003 ▶); Gelderblom & Sparreboom (2006 ▶). For the biological activity of quinolines, see: Ewesuedo *et al.* (2001 ▶); Ishida & Asao (2002 ▶); Kouznetsov *et al.* (2006 ▶); Li *et al.* (2006 ▶); Ohyama *et al.* (1999 ▶); Priel *et al.* (1991 ▶); Twelves *et al.* (1999 ▶); Martínez & Chacón-García (2005 ▶); Pommier (2006 ▶).
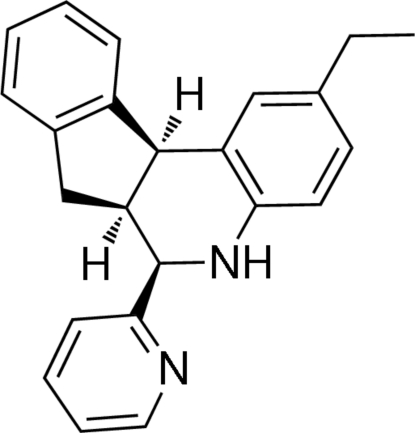


## Experimental

### 

#### Crystal data

C_23_H_22_N_2_
*M* *_r_* = 326.43
Monoclinic, 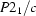

*a* = 13.241 (4) Å
*b* = 15.801 (4) Å
*c* = 8.789 (2) Å
β = 101.168 (6)°
*V* = 1804.0 (8) Å^3^
*Z* = 4
Mo *K*α radiation
μ = 0.07 mm^−1^
*T* = 293 K
0.30 × 0.28 × 0.26 mm

#### Data collection

Rigaku AFC7S Mercury diffractometer
Absorption correction: multi-scan (Jacobson, 1998 ▶) *T*_min_ = 0.971, *T*_max_ = 0.981
20284 measured reflections
3688 independent reflections
2420 reflections with *I* > 2σ(*I*)
*R*_int_ = 0.044
Standard reflections: 0

#### Refinement

*R*[*F* ^2^ > 2σ(*F* ^2^)] = 0.058
*wR*(*F* ^2^) = 0.157
*S* = 1.07
3688 reflections
226 parameters
H-atom parameters constrained
Δρ_max_ = 0.25 e Å^−3^
Δρ_min_ = −0.20 e Å^−3^

### 

Data collection: *CrystalClear* (Rigaku, 2002 ▶); cell refinement: *CrystalClear*; data reduction: *CrystalClear*; program(s) used to solve structure: *SHELXTL-NT* (Sheldrick, 2008 ▶); program(s) used to refine structure: *SHELXTL-NT*; molecular graphics: *SHELXTL-NT* and *DIAMOND* (Brandenburg, 1998 ▶); software used to prepare material for publication: *SHELXTL-NT* and *PLATON* (Spek, 2009 ▶).

## Supplementary Material

Crystal structure: contains datablocks I. DOI: 10.1107/S1600536810005805/tk2615sup1.cif

Structure factors: contains datablocks I. DOI: 10.1107/S1600536810005805/tk2615Isup2.hkl

Additional supplementary materials:  crystallographic information; 3D view; checkCIF report

## Figures and Tables

**Table 1:** Hydrogen-bond geometry (Å, °) *Cg*4 is the centroid of the C4–C9 ring.

*D*—H⋯*A*	*D*—H	H⋯*A*	*D*⋯*A*	*D*—H⋯*A*
N1—H1*N*⋯N2^i^	0.87	2.53	3.345 (2)	157
C20—H20⋯N1	0.93	2.51	2.825 (3)	100
C14—H14⋯*Cg*4^ii^	0.93	2.74	3.611 (2)	153

Symmetry codes: (i) 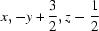; (ii) 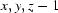.

**Fig. 1.**
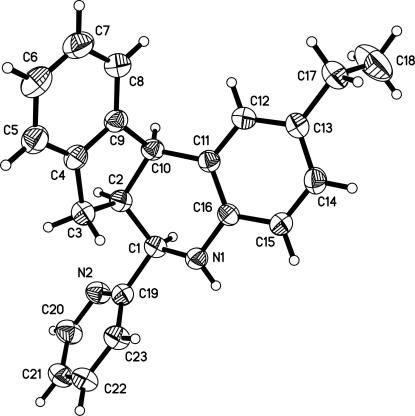
Molecular structure of (I) showing the atom-labelling scheme. Displacement ellipsoids are drawn at the 50% probability level. H atoms are represented by circles of arbitrary size.

**Fig. 2.**
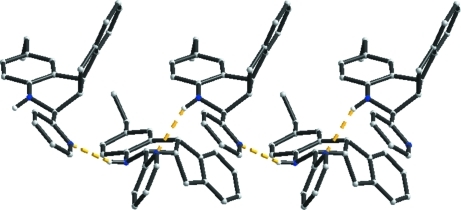
View of a one-dimensional ribbon aligned along the c axis, generated by N—H···N hydrogen bonds. Intermolecular hydrogen bonds are indicated by dashed lines. H atoms not involved in hydrogen bonding have been omitted for clarity.

## supplementary crystallographic information

### Comment

Within the quinoline family, polycyclic analogues are the most relevant compounds due to their broad potential as antitumoral agents (Gelderblom & Sparreboom, 2006; Denny & Baguley, 2003). Since the discovery of camptothecin, a natural topopisomerase (topo) I inhibitor (Pommier, 2006; Priel *et al.*, 1991), a constant search for new compounds with the ability for inhibit the topoisomerases I/II enzymes has been undertaken (Li *et al.*, 2006; Martínez & Chacón-García, 2005). The compound (6-[2-(dimethylamino)ethylamino]-3-hydroxy-7*H*-indeno[2,1-*c*] quinolin-7-one dihydrochloride (known as TAS-103) presents potent cytotoxicity in different leukemia lines (Twelves *et al.*, 1999; Ohyama *et al.*, 1999). The exhibited anti-cancer activity is due to its ability to function as a dual inhibitor of both topo I/II, and it has been investigated in clinical studies in recent years (Ewesuedo *et al.*, 2001; Ishida & Asao, 2002).

In our preliminary studies of TAS-103 analogues, we have developed the synthesis (using the imino Diels Alder reaction) and studied the biological activity of the 6-α-pyridinyl- tetrahydro)indeno[2,1-*c*]quinolines (Kouznetsov *et al.*, 2006). It was found that these compounds were active against MCF-7, H-460 and SF-268 cancer cell lines making them potential anti-cancer agents (Kouznetsov *et al.*, 2006).

In order to obtain detailed information on its molecular conformation and the stereochemistry of the reaction, in this contribution, the molecular structure of the title compound, (I), is described. The structural analysis indicated (I) exists in the *cis*-form as a unique regio- and diastereo-isomer (Fig. 1). The tetrahydropyridine ring adopts a half-chair conformation and the indene ring displays an envelope configuration. The crystal packing of (I) consists of one-dimensional zigzag ribbons that run along the *c* direction and linked *via* N—H···N hydrogen bonding interactions (Fig. 2 & Table 1).

### Experimental

A mixture of aryl amine (3.6 mmol) and α-pyridinecarboxyaldehyde (4.0 mmol) in anhydrous CH_3_CN (15 ml) was stirred at room temperature for 30 min after which BF_3_.OEt_2_ (3.6 mmol) was added. Over a period of 20 min, an acetonitrile solution (10 ml) of indene (4.0 mmol) was added dropwise. The resulting mixture was stirred at 343 K for 5 h. After completion of the reaction, as indicated by TLC, the reaction mixture was diluted with water (30 ml) and extracted with ethyl acetate (3 *x* 15 ml). The organic layer was separated and dried (Na_2_SO_4_), concentrated *in vacuo*, and the resulting product was purified by column chromatography (silica gel, petroleum ether: EtOAc) to afford pure (I) as a colorless solid, mp 424–425 K (yield 43%). This compound was recrystallized by slow evaporation from the solvent mixture, hexane-ethyl acetate.

### Refinement

All H atoms were placed in geometrically idealized positions and constrained to ride on their parent atoms, with C—H distances of 0.93 (aromatic) and 0.96 Å (methyl), and with *U*_iso_(H) = 1.5 (1.2 for aromatic-H atoms) times *U*_eq_(C). The low completeness ratio is due to the experimental setup whereby the equipment has a χ circle and an added area detector (four-circle diffractometer modified with a CCD). This precludes the collection of some regions of reciprocal lattice space and lowers the completeness. In order to compensate, additional redundant data were measured.

### Crystal data

**Table:**

C_23_H_22_N_2_	*F*(000) = 696
*M**_r_* = 326.43	*D*_x_ = 1.202 Mg m^−^^3^
Monoclinic, *P*2_1_/*c*	Mo *K*α radiation, λ = 0.71073 Å
Hall symbol: -P 2ybc	Cell parameters from 11041 reflections
*a* = 13.241 (4) Å	θ = 1.6–27.7°
*b* = 15.801 (4) Å	µ = 0.07 mm^−^^1^
*c* = 8.789 (2) Å	*T* = 293 K
β = 101.168 (6)°	Block, yellow
*V* = 1804.0 (8) Å^3^	0.30 × 0.28 × 0.26 mm
*Z* = 4	

### Data collection

**Table:**

Rigaku AFC7S Mercury diffractometer	3688 independent reflections
Radiation source: Normal-focus sealed tube	2420 reflections with *I* > 2σ(*I*)
graphite	*R*_int_ = 0.044
ω scans	θ_max_ = 28.0°, θ_min_ = 2.0°
Absorption correction: multi-scan (Jacobson, 1998)	*h* = −15→15
*T*_min_ = 0.971, *T*_max_ = 0.981	*k* = −18→20
20284 measured reflections	*l* = −11→11

### Refinement

**Table:**

Refinement on *F*^2^	Primary atom site location: structure-invariant direct methods
Least-squares matrix: full	Secondary atom site location: difference Fourier map
*R*[*F*^2^ > 2σ(*F*^2^)] = 0.058	Hydrogen site location: inferred from neighbouring sites
*wR*(*F*^2^) = 0.157	H-atom parameters constrained
*S* = 1.07	*w* = 1/[σ^2^(*F*_o_^2^) + (0.0698*P*)^2^ + 0.2578*P*] where *P* = (*F*_o_^2^ + 2*F*_c_^2^)/3
3688 reflections	(Δ/σ)_max_ < 0.001
226 parameters	Δρ_max_ = 0.25 e Å^−^^3^
0 restraints	Δρ_min_ = −0.20 e Å^−^^3^

### Special details

**Table:**

Geometry. All e.s.d.'s (except the e.s.d. in the dihedral angle between two l.s. planes) are estimated using the full covariance matrix. The cell e.s.d.'s are taken into account individually in the estimation of e.s.d.'s in distances, angles and torsion angles; correlations between e.s.d.'s in cell parameters are only used when they are defined by crystal symmetry. An approximate (isotropic) treatment of cell e.s.d.'s is used for estimating e.s.d.'s involving l.s. planes.
Refinement. Refinement of *F*^2^ against ALL reflections. The weighted *R*-factor *wR* and goodness of fit *S* are based on *F*^2^, conventional *R*-factors *R* are based on *F*, with *F* set to zero for negative *F*^2^. The threshold expression of *F*^2^ > σ(*F*^2^) is used only for calculating *R*-factors(gt) *etc*. and is not relevant to the choice of reflections for refinement. *R*-factors based on *F*^2^ are statistically about twice as large as those based on *F*, and *R*- factors based on ALL data will be even larger.

### Fractional atomic coordinates and isotropic or equivalent isotropic displacement parameters (Å^2^)

**Table:**

	*x*	*y*	*z*	*U*_iso_*/*U*_eq_	
N1	0.02307 (11)	0.64024 (9)	0.43799 (17)	0.0490 (4)	
H1N	−0.0192	0.6589	0.3565	0.059*	
N2	−0.13358 (12)	0.73555 (10)	0.68890 (19)	0.0562 (4)	
C1	0.00785 (13)	0.68474 (11)	0.5771 (2)	0.0460 (4)	
H1	0.0242	0.7447	0.5668	0.055*	
C2	0.07995 (13)	0.64878 (11)	0.7193 (2)	0.0458 (4)	
H2	0.0741	0.6845	0.8084	0.055*	
C3	0.05427 (14)	0.55713 (11)	0.7591 (2)	0.0528 (5)	
H3A	0.0073	0.5563	0.8311	0.063*	
H3B	0.0237	0.5261	0.6663	0.063*	
C4	0.15662 (15)	0.52035 (11)	0.8317 (2)	0.0499 (5)	
C5	0.17744 (17)	0.44613 (13)	0.9155 (2)	0.0636 (6)	
H5	0.1240	0.4127	0.9366	0.076*	
C6	0.2788 (2)	0.42241 (15)	0.9675 (3)	0.0744 (7)	
H6	0.2934	0.3721	1.0223	0.089*	
C7	0.35794 (19)	0.47219 (16)	0.9391 (3)	0.0766 (7)	
H7	0.4257	0.4555	0.9753	0.092*	
C8	0.33764 (16)	0.54720 (14)	0.8568 (2)	0.0653 (6)	
H8	0.3915	0.5811	0.8387	0.078*	
C9	0.23611 (14)	0.57118 (11)	0.8019 (2)	0.0485 (5)	
C10	0.19475 (13)	0.64765 (11)	0.7040 (2)	0.0456 (4)	
H10	0.2292	0.6992	0.7495	0.055*	
C11	0.20897 (13)	0.63816 (10)	0.5372 (2)	0.0434 (4)	
C12	0.30732 (15)	0.63126 (11)	0.5033 (2)	0.0523 (5)	
H12	0.3636	0.6365	0.5845	0.063*	
C13	0.32582 (15)	0.61707 (12)	0.3557 (2)	0.0540 (5)	
C14	0.24015 (16)	0.60999 (12)	0.2362 (2)	0.0545 (5)	
H14	0.2494	0.5997	0.1356	0.065*	
C15	0.14227 (14)	0.61795 (11)	0.2647 (2)	0.0485 (5)	
H15	0.0864	0.6128	0.1828	0.058*	
C16	0.12446 (13)	0.63354 (10)	0.4138 (2)	0.0427 (4)	
C17	0.43476 (17)	0.60998 (15)	0.3268 (3)	0.0731 (7)	
H17A	0.4802	0.5943	0.4230	0.088*	
H17B	0.4371	0.5649	0.2526	0.088*	
C18	0.4737 (2)	0.6887 (2)	0.2677 (5)	0.1291 (13)	
H18A	0.5427	0.6798	0.2520	0.194*	
H18B	0.4734	0.7334	0.3416	0.194*	
H18C	0.4303	0.7040	0.1710	0.194*	
C19	−0.10323 (13)	0.67742 (11)	0.5962 (2)	0.0459 (4)	
C20	−0.16803 (15)	0.61310 (12)	0.5276 (2)	0.0562 (5)	
H20	−0.1446	0.5731	0.4650	0.067*	
C21	−0.26739 (16)	0.60932 (13)	0.5534 (3)	0.0620 (6)	
H21	−0.3120	0.5669	0.5082	0.074*	
C22	−0.29921 (16)	0.66882 (15)	0.6463 (3)	0.0667 (6)	
H22	−0.3660	0.6680	0.6650	0.080*	
C23	−0.23034 (16)	0.73019 (14)	0.7117 (3)	0.0667 (6)	
H23	−0.2525	0.7703	0.7755	0.080*	

### Atomic displacement parameters (Å^2^)

**Table:**

	*U*^11^	*U*^22^	*U*^33^	*U*^12^	*U*^13^	*U*^23^
N1	0.0429 (9)	0.0609 (9)	0.0431 (9)	0.0008 (7)	0.0077 (7)	−0.0047 (7)
N2	0.0480 (10)	0.0581 (10)	0.0636 (11)	0.0033 (7)	0.0136 (8)	−0.0097 (8)
C1	0.0445 (11)	0.0427 (9)	0.0513 (11)	−0.0007 (7)	0.0103 (8)	−0.0045 (8)
C2	0.0460 (11)	0.0466 (10)	0.0451 (10)	−0.0006 (7)	0.0091 (8)	−0.0094 (8)
C3	0.0511 (12)	0.0547 (11)	0.0533 (11)	−0.0027 (8)	0.0122 (9)	0.0011 (9)
C4	0.0565 (12)	0.0515 (11)	0.0408 (10)	0.0000 (8)	0.0067 (8)	−0.0038 (8)
C5	0.0707 (15)	0.0608 (13)	0.0563 (12)	−0.0049 (10)	0.0051 (11)	0.0056 (10)
C6	0.0868 (18)	0.0677 (14)	0.0625 (14)	0.0120 (13)	−0.0013 (13)	0.0099 (11)
C7	0.0646 (16)	0.0928 (17)	0.0676 (15)	0.0176 (13)	0.0005 (12)	0.0117 (13)
C8	0.0509 (13)	0.0838 (15)	0.0593 (13)	0.0032 (10)	0.0056 (10)	0.0078 (11)
C9	0.0475 (12)	0.0563 (11)	0.0402 (10)	0.0008 (8)	0.0050 (8)	−0.0065 (8)
C10	0.0439 (11)	0.0461 (10)	0.0461 (10)	−0.0033 (7)	0.0066 (8)	−0.0066 (8)
C11	0.0439 (11)	0.0418 (9)	0.0445 (10)	−0.0030 (7)	0.0086 (8)	−0.0019 (7)
C12	0.0436 (12)	0.0595 (12)	0.0526 (12)	−0.0034 (8)	0.0065 (9)	−0.0006 (9)
C13	0.0498 (12)	0.0606 (12)	0.0540 (12)	0.0000 (8)	0.0164 (10)	0.0008 (9)
C14	0.0591 (13)	0.0595 (12)	0.0478 (11)	−0.0007 (9)	0.0178 (10)	−0.0022 (9)
C15	0.0489 (12)	0.0524 (11)	0.0430 (10)	−0.0017 (8)	0.0060 (9)	−0.0009 (8)
C16	0.0423 (11)	0.0394 (9)	0.0471 (10)	−0.0024 (7)	0.0107 (8)	−0.0004 (7)
C17	0.0557 (14)	0.0962 (17)	0.0727 (15)	0.0020 (11)	0.0255 (12)	−0.0024 (13)
C18	0.085 (2)	0.129 (3)	0.188 (4)	−0.0048 (18)	0.064 (2)	0.038 (3)
C19	0.0462 (11)	0.0439 (10)	0.0474 (10)	0.0035 (8)	0.0083 (8)	−0.0001 (8)
C20	0.0514 (12)	0.0529 (11)	0.0651 (13)	−0.0019 (8)	0.0134 (10)	−0.0089 (9)
C21	0.0496 (13)	0.0631 (13)	0.0738 (15)	−0.0089 (9)	0.0129 (11)	−0.0028 (11)
C22	0.0467 (12)	0.0813 (15)	0.0751 (15)	−0.0015 (11)	0.0192 (11)	−0.0010 (12)
C23	0.0532 (14)	0.0742 (14)	0.0765 (15)	0.0050 (10)	0.0218 (11)	−0.0149 (11)

### Geometric parameters (Å, °)

**Table:**

N1—C16	1.403 (2)	C10—H10	0.9800
N1—C1	1.458 (2)	C11—C12	1.395 (3)
N1—H1N	0.8700	C11—C16	1.401 (2)
N2—C23	1.337 (2)	C12—C13	1.384 (3)
N2—C19	1.340 (2)	C12—H12	0.9300
C1—C19	1.517 (2)	C13—C14	1.393 (3)
C1—C2	1.528 (2)	C13—C17	1.516 (3)
C1—H1	0.9800	C14—C15	1.373 (3)
C2—C3	1.543 (2)	C14—H14	0.9300
C2—C10	1.552 (2)	C15—C16	1.397 (2)
C2—H2	0.9800	C15—H15	0.9300
C3—C4	1.499 (3)	C17—C18	1.479 (3)
C3—H3A	0.9700	C17—H17A	0.9700
C3—H3B	0.9700	C17—H17B	0.9700
C4—C5	1.384 (3)	C18—H18A	0.9600
C4—C9	1.389 (3)	C18—H18B	0.9600
C5—C6	1.383 (3)	C18—H18C	0.9600
C5—H5	0.9300	C19—C20	1.390 (3)
C6—C7	1.371 (3)	C20—C21	1.379 (3)
C6—H6	0.9300	C20—H20	0.9300
C7—C8	1.387 (3)	C21—C22	1.364 (3)
C7—H7	0.9300	C21—H21	0.9300
C8—C9	1.390 (3)	C22—C23	1.378 (3)
C8—H8	0.9300	C22—H22	0.9300
C9—C10	1.522 (2)	C23—H23	0.9300
C10—C11	1.521 (2)		
			
C16—N1—C1	117.08 (14)	C12—C11—C16	118.00 (17)
C16—N1—H1N	112.5	C12—C11—C10	120.53 (16)
C1—N1—H1N	110.8	C16—C11—C10	121.44 (16)
C23—N2—C19	117.16 (17)	C13—C12—C11	123.67 (18)
N1—C1—C19	110.52 (14)	C13—C12—H12	118.2
N1—C1—C2	109.93 (14)	C11—C12—H12	118.2
C19—C1—C2	110.28 (14)	C12—C13—C14	116.96 (18)
N1—C1—H1	108.7	C12—C13—C17	121.04 (18)
C19—C1—H1	108.7	C14—C13—C17	122.00 (18)
C2—C1—H1	108.7	C15—C14—C13	120.98 (18)
C1—C2—C3	113.82 (14)	C15—C14—H14	119.5
C1—C2—C10	113.60 (14)	C13—C14—H14	119.5
C3—C2—C10	105.75 (14)	C14—C15—C16	121.60 (17)
C1—C2—H2	107.8	C14—C15—H15	119.2
C3—C2—H2	107.8	C16—C15—H15	119.2
C10—C2—H2	107.8	C15—C16—C11	118.71 (17)
C4—C3—C2	103.86 (15)	C15—C16—N1	119.70 (16)
C4—C3—H3A	111.0	C11—C16—N1	121.53 (16)
C2—C3—H3A	111.0	C18—C17—C13	113.9 (2)
C4—C3—H3B	111.0	C18—C17—H17A	108.8
C2—C3—H3B	111.0	C13—C17—H17A	108.8
H3A—C3—H3B	109.0	C18—C17—H17B	108.8
C5—C4—C9	120.69 (18)	C13—C17—H17B	108.8
C5—C4—C3	128.74 (18)	H17A—C17—H17B	107.7
C9—C4—C3	110.56 (16)	C17—C18—H18A	109.5
C6—C5—C4	119.1 (2)	C17—C18—H18B	109.5
C6—C5—H5	120.5	H18A—C18—H18B	109.5
C4—C5—H5	120.5	C17—C18—H18C	109.5
C7—C6—C5	120.8 (2)	H18A—C18—H18C	109.5
C7—C6—H6	119.6	H18B—C18—H18C	109.5
C5—C6—H6	119.6	N2—C19—C20	122.16 (17)
C6—C7—C8	120.5 (2)	N2—C19—C1	115.24 (15)
C6—C7—H7	119.8	C20—C19—C1	122.56 (16)
C8—C7—H7	119.8	C21—C20—C19	119.26 (18)
C7—C8—C9	119.4 (2)	C21—C20—H20	120.4
C7—C8—H8	120.3	C19—C20—H20	120.4
C9—C8—H8	120.3	C22—C21—C20	118.92 (19)
C4—C9—C8	119.61 (19)	C22—C21—H21	120.5
C4—C9—C10	111.27 (16)	C20—C21—H21	120.5
C8—C9—C10	129.09 (18)	C21—C22—C23	118.6 (2)
C11—C10—C9	111.60 (14)	C21—C22—H22	120.7
C11—C10—C2	113.02 (14)	C23—C22—H22	120.7
C9—C10—C2	102.20 (14)	N2—C23—C22	123.90 (19)
C11—C10—H10	109.9	N2—C23—H23	118.1
C9—C10—H10	109.9	C22—C23—H23	118.1
C2—C10—H10	109.9		

### Hydrogen-bond geometry (Å, °)

**Table:**

Cg4 is the centroid of the C4–C9 ring.

**Table:**

*D*—H···*A*	*D*—H	H···*A*	*D*···*A*	*D*—H···*A*
N1—H1N···N2^i^	0.87	2.53	3.345 (2)	157
C20—H20···N1	0.93	2.51	2.825 (3)	100
C14—H14···Cg4^ii^	0.93	2.74	3.611 (2)	153

Symmetry codes: (i) *x*, −*y*+3/2, *z*−1/2;  (ii) *x*, *y*, *z*−1.
